# Local-Based Semantic Navigation on a Networked Representation of Information

**DOI:** 10.1371/journal.pone.0043694

**Published:** 2012-08-24

**Authors:** José A. Capitán, Javier Borge-Holthoefer, Sergio Gómez, Juan Martinez-Romo, Lourdes Araujo, José A. Cuesta, Alex Arenas

**Affiliations:** 1 Departament d’Enginyeria Informàtica i Matemàtiques, Universitat Rovira i Virgili, Tarragona, Spain; 2 Centro de Astrobiologa, Consejo Superior de Investigaciones Científicas-INTA, Torrejón de Ardoz, Madrid, Spain; 3 Grupo Interdisciplinar de Sistemas Complejos, Madrid, Spain; 4 Instituto de Biocomputación y Física de Sistemas Complejos, Universidad de Zaragoza, Zaragoza, Spain; 5 Departamento de Lenguajes y Sistemas Informáticos, Universidad Nacional de Educación a Distancia, Madrid, Spain; 6 Departamento de Matemáticas, Escuela Politécnica Superior, Universidad Carlos III de Madrid, Leganés, Madrid, Spain; University of Maribor, Slovenia

## Abstract

The size and complexity of actual networked systems hinders the access to a global knowledge of their structure. This fact pushes the problem of navigation to suboptimal solutions, one of them being the extraction of a coherent map of the topology on which navigation takes place. In this paper, we present a Markov chain based algorithm to tag networked terms according only to their topological features. The resulting tagging is used to compute similarity between terms, providing a map of the networked information. This map supports local-based navigation techniques driven by similarity. We compare the efficiency of the resulting paths according to their length compared to that of the shortest path. Additionally we claim that the path steps towards the destination are semantically coherent. To illustrate the algorithm performance we provide some results from the Simple English Wikipedia, which amounts to several thousand of pages. The simplest greedy strategy yields over an 80% of average success rate. Furthermore, the resulting content-coherent paths most often have a cost between one- and threefold compared to shortest-path lengths.

## Introduction

Efficient network navigation is a challenging puzzle that has many sides to it. From a practical point of view, successful navigation is important for example in human mobility [1], [2] or social networks [3], but also on the Internet, regarding content-sharing applications and search engines [4], or packet routing at the Autonomous Systems level [5]. On more theoretical grounds it has inspired research on *navigability*, or the minimum features a structure must exhibit to guarantee efficient navigation on it [6], [7]. It also poses an algorithmic problem which reduces to the design of heuristics handling a certain amount of knowledge about the underlying topology. The problem even exhibits a sociopsychological dimension, as the seminal work by Milgram [8], [9] illustrates. Of course, the situation in which the nodes of a network have at hand a coherent view of the global topology trivially renders optimal navigation –the target can *always* be achieved with the *smallest* amount of hops. But most often this is not the case. Any other scenario will yield a suboptimal outcome, depending on the ability of the heuristics and the quality of the map.

A map is a more or less cogent representation retaining information from the network on which navigation takes place. Many works in the literature focus on algorithmic design, assuming that some kind of map –“a reference frame”– is already available to the navigator. Then, knowing that “I must move eastwards” entails I have a notion of where the East lies [1]. In a different fashion, knowing that I should move to a better connected street (autonomous system, airport, etc.) entails that I have a certain notion of the topology around me [10], [11]. The success of Milgram’s letter-passing experiment relies on a mixture of the previous two cases –a cognitive ability encoding both spatial representation and the knowledge of the agent’s surrounding social network. On the other hand, only a few works approach navigation facing the problem of building a map from scratch. The work by Boguñá and collaborators relies on different geometric embeddings from which hidden space metrics emerge and allow for greedy, decentralized navigation [2], [5]. Similarly, Erola *et al.* [12], [13] capitalize on the properties of Singular Value Decomposition to obtain a multidimensional projection of a connectivity matrix [14], which can ultimately be used as a guiding map.

**Figure 1:**
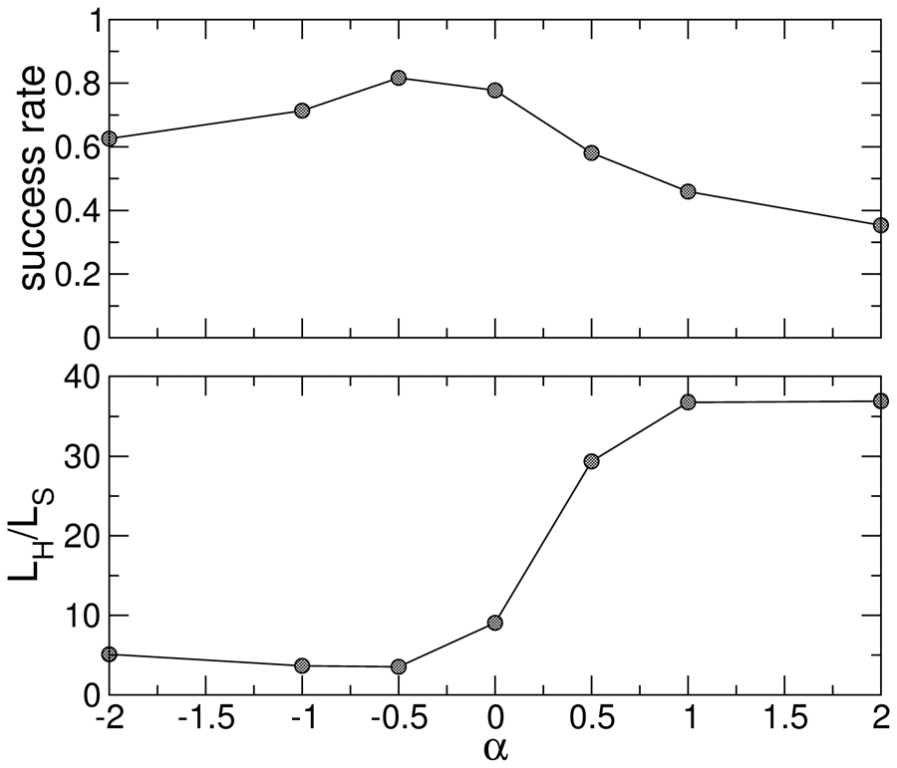
Success ratio (upper pannel) and length ratio (lower panel) of semantic paths reaching the destination as a function of the weighting scheme 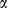.

In this work we confront the building of a map in a different manner. We rely on the intuition that the way to attain a reliable representation of a structure is to (randomly) walk it. Random walks have been largely exploited in the complex network literature as a fundamental dynamic process [15] which has proved useful to tackle the issue of community detection [16], [17] or as a way to approach search and transport problems [18], to mention just a few. Our proposal amounts to exploring the network using random walks, and compares pairs of nodes according to their relative view of the whole network according to the paths emerging from the diffusion of walkers. The algorithm performing such a task is called Random Inheritance Model (RIM) [19]. RIM stems out of the family of “spreading activation” algorithms which were put forward in the field of Cognitive Science as early as the 1960 s [20]–[22]. “Spreading activation” may well be seen as the mechanism upon which semantics emerge, thus RIM –or, in general, the random dynamics behind it– can be regarded as a general tool to extract a detailed “reference frame” for navigators. The key idea is that nodes that *observe* the same perspective of the rest of the network are *similar* to each other. In the case of words we show that this similarity indicates that they are semantically related.

The use of RIM to obtain an efficiently navigable map depends on having an underlying networked structure. Because the map is, furthermore, semantically sound, the easiest way to evidence it is to work on a network involving language. A statistically robust way to obtain a network of words is to build a co-occurrence graph from text sources, see for example Ref. [23]. However, we construct the semantic similarity map obtained from the complete Simple English Wikipedia (SEW from now on), which can be naturally modeled as a network and contains over 50,000 pages. After building up the semantically sensitive map, we show its potential proposing a local-based semantic navigation. Semantic paths between pairs of words are obtained according to a Milgram-like navigation: given an accurate map, the navigator just needs to check who, in its own neighborhood, has a greater similarity to the target, and move accordingly. To evaluate this navigation we compare the efficiency of the resulting paths according to their length compared to that of the shortest path. Secondarily, we illustrate with examples the semantic coherence in the path steps towards the destination. Imagine, for example, that we want to find a path between two pages of SEW such as Norway Iowa and Yuri Gagarin. The shortest-length path (which implies global information of the connectivity) from source to target is: Norway Iowa 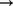 United States 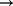 January 1 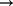 March 27 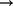 Yuri Gagarin. Note that the resulting path is pretty uninformative by itself. However, our approach produces Norway Iowa 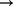 United States 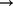 History of the United States 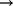 Moon 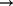 Astronaut 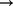 Yuri Gagarin, a path comprising local information only. In the latter navigation we learn that Yuri Gagarin was an astronaut, and that the US were involved in the space race to achieve the first human-trip to the Moon.

**Figure 2:**
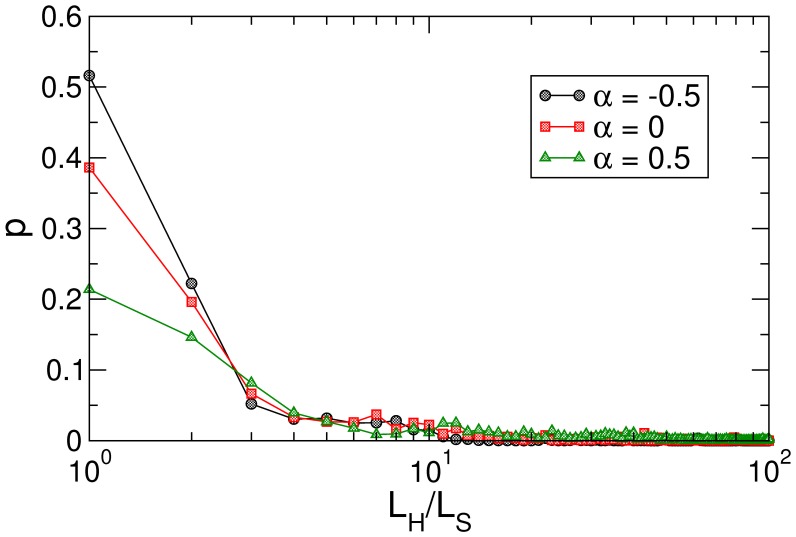
Proportion of successful paths, navigation attempts which reach the target as a function of their length cost compared to shortest paths 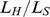.

Our results, which are –as stated above– suboptimal, are comparable to shortest paths and suggest the use of this navigation technique to complement search in Web browsers, recommendation systems, and information discovery.

## Methods

### Building Up the Similarity Map

Given a networked representation of information, our aim in this work is to derive a map that permits a coherent exploration of the network through local navigation on it. To this end, we will extract similarity relationships between nodes from the track of a dynamical process displayed over the network. Recent works have pointed out the ability of random walkers to explore the topological structure of networks [15], [24], [25], and its relation with cognitive abilities [26]. In addition, random walkers can serve as a convenient tool to unveil categorical relationships out of the network. This is due to the fact that random walkers are the simplest dynamical processes capable of revealing local neighborhoods of nodes in which walkers get persistently trapped, and these groups are expected to retain significant meta-similitude relationships. This fact, together with an inheritance mechanism aimed to reinforce the similarities within local vicinities of nodes, constitute the basis of the Random Inheritance Model [19].

**Figure 3:**
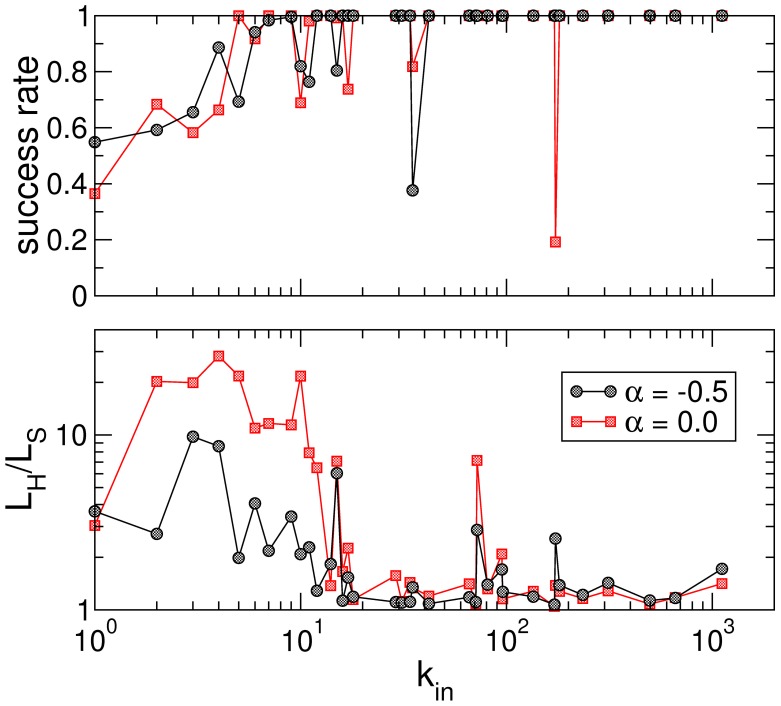
Success ratio of semantic paths reaching the destination (upper panel), and length ratio compared (lower panel) as a function of target’s accessibility represented by its in-degree 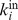.

RIM proceeds as follows. First, every node 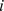 in the network is tagged with an initial, 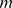-dimensional feature vector 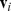, 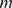 being the size (number of nodes) of the network. This vector is initially chosen such that its 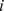-th entry is equal to one and the remaining entries are zero, i.e., vectors are orthogonal in the canonical basis to avoid any initial bias. The second step consists in launching random walks of a fixed length 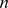 from every node in the network. The inheritance mechanism modifies features depending on the exploration of the network performed by the walker. Let 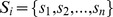 be the set of nodes visited by a walker starting from 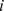. Then the new feature vector 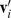 is computed by averaging the feature vectors over the set of visited nodes,
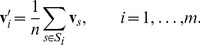
(1)


**Table 1:** Comparison between semantic navigation and shortest path for a sample of source and target pairs of words.

*Semantic Navigation*	*Shortest Path*	*Semantic Navigation*	*Shortest Path*
Microsoft_Access	Microsoft_Access	Pandora	Pandora
Computer_program	Database	Jar	Wine
Application	Leaf	Leyden_jar	United_States
Human_body	Biology	Capacitor	Electronics
Biology	Evolutionary_biology	Inductor	Electrical_circuit
Evolutionary_biology		Electrical_circuit	
Norway_Iowa	Norway_Iowa	Wii_Sports	Wii_Sports
United_States	United_States	Tennis	Wii
U_States_History	January_1	England	2006
Moon	March_27	Protestantism	Good_Friday
Astronaut	Yuri_Gagarin	Paul_the_Apostle	Judas_Iscariot
Yuri_Gagarin		Judas_Iscariot	
Gerardus_Mercator	Gerardus_Mercator	Oxfam	Oxfam
Atlas	Atlas	United_Kingdom	Canada
Google_Maps	Rome	United_States	July_1
Satellite	NASA	Computer	Windows_2000
Sputnik	Space_Race	Operating_system	Novell
U.S.S.R.		Linux	OpenSuSE
Cold_War		SuSE	
Space_Race		OpenSuSE	
Electricity	Electricity	Liza_Minnelli	Liza_Minnelli
Oil	Metal	United_States	June_24
Maize	Zinc	Forest	July_1
Grain	Cereal	Rainforest	Evolution
Oat	Cheerios	Bird	Genetic_drift
Cereal		Evolution	
Cheerios		Genetic_drift	
Space_Race	Space_Race	Taco_Bell	Taco_Bell
United_States	1957	United_States	June_9
Computer	1960 s	U_States_History	December_21
Operating_system	UNIX	Roaring_Twenties	F_Scott_Fitzgerald
UNIX		F_Scott_Fitzgerald	The_Great_Gatsby
		The_Great_Gatsby	

In some cases the shortest path led to degenerated chains (one of which is shown here).

This way nodes ‘inherit’ the features of all nodes visited along the path. Note that final values are computed after completion of the inheritance for every node (synchronous update of the feature vectors). Finally a map, under the form of a similarity matrix 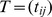, is obtained. This matrix contains weighted values for each pair of nodes, which result from projecting all pairs of updated vectors (cosine similarity),
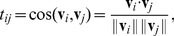
(2)where 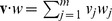 stands for the Euclidean dot product and 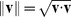 is its associated norm.

**Figure 4:**
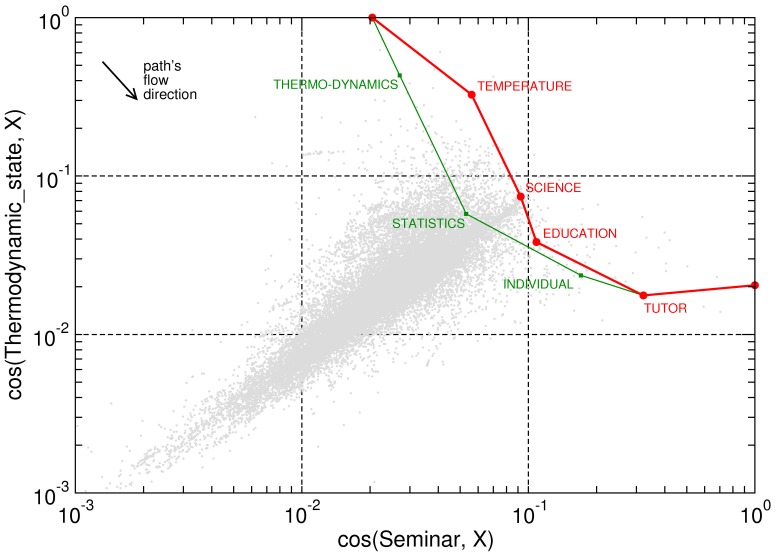
Similarity to target (Thermodynamic_State) vs. similarity to source (Seminar). Semantic navigation (red trajectory) behaves similarly to shortest path (green trajectory): there are only two degenerated shortest paths and one of them coincides with the semantic path. This example shows that semantic navigation efficiency can be optimal in some cases, because the number of jumps equals to that of shortest path navigation. Conversely, shortest paths sometimes can (accidentally) yield coherent paths in terms of meaning. The remaining similarity pairs with the rest of the network are depicted as a scatter plot.

The similarity matrix can be calculated in terms of the transition probability matrix 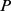 of the random walk used to explore the network. The 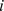-th row of matrix 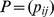 specifies the probability 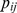 for the walker to jump from 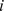 to any of its neighbors 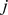. If the underlying network is weighted, setting up the transition probability matrix amounts to normalizing the weights so that the out-strength of any node 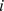 (i.e., the sum of weights for all directed links connecting 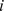 with its neighbors) is equal to 1. If the network is unweighted, all connections of any node are equally relevant. In this case, a common proposal in the field of complex networks amounts to weighting links according to the importance (in terms of degree) of the nodes they connect [27]. For undirected networks, normalized weights take the form
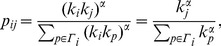
(3)

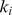
 being the degree of node 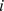, 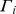 the set of 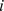
‘s neighbors, and 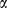 a tuning parameter to give more or less importance to the local connectivity of nodes. We refer the reader to the following subsection for details on how these ideas can be extended to set up the weights of directed networks like SEW. Note that the normalizing factor in the denominator transforms the matrix of weights into a stochastic matrix 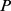, which in turn allows us to describe the algorithm in terms of a Markov chain.

**Figure 5:**
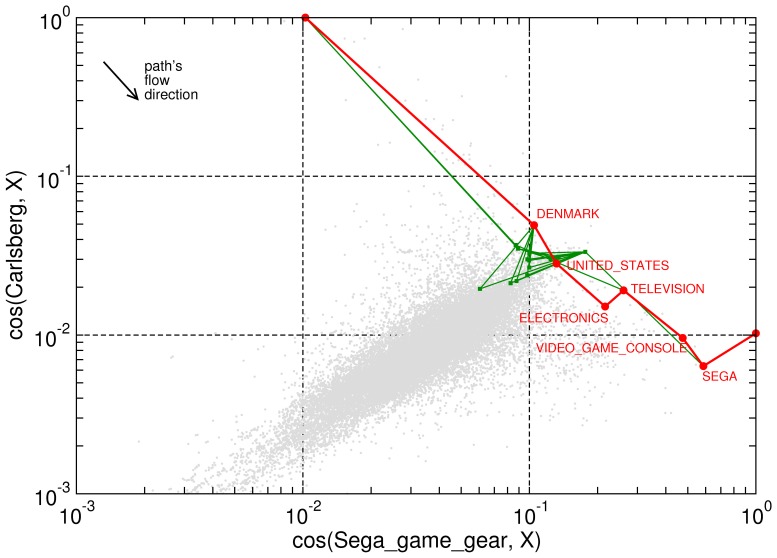
Similarity to target (Carlsberg) vs. similarity to source (Sega_Game_Gear). In this example, our semantic path (red) is comprised by 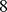 jumps whereas shortest paths (green) involve 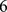 steps (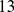-fold degenerated). However, a slight efficiency loss can be compensated by a truly coherent path. Observe how the shortest path decreases its similarity to the target at some intermediate points. At these points, shortest paths navigate through hubs (like September_7 or 1999) which exhibit shallow similarities with source and target, but help to reach the target in a small number of steps. The remaining similarity pairs with the rest of nodes are depicted as a scatter plot.

The entry 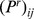 of the 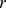-th power of 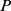 has a very important meaning for our purposes. It stands for the probability of hitting node 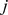, starting from 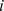, in exactly 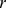 steps. In practice, this means that if we perform random walks of length 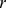, after averaging over many realizations the frequency of visiting node 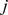 (starting from 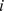) will be 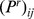. According to Eq. (1), the inheritance process yields, in this scenario, feature vectors that are simply the rows of the matrix
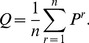
(4)


Similarity between nodes is calculated as the cosine [cf. Eq. (2)] of the angle between each pair of row vectors of matrix 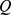. Thus, the similarity matrix 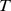 is now ready to be used for navigational purposes over the original network.

**Figure 6:**
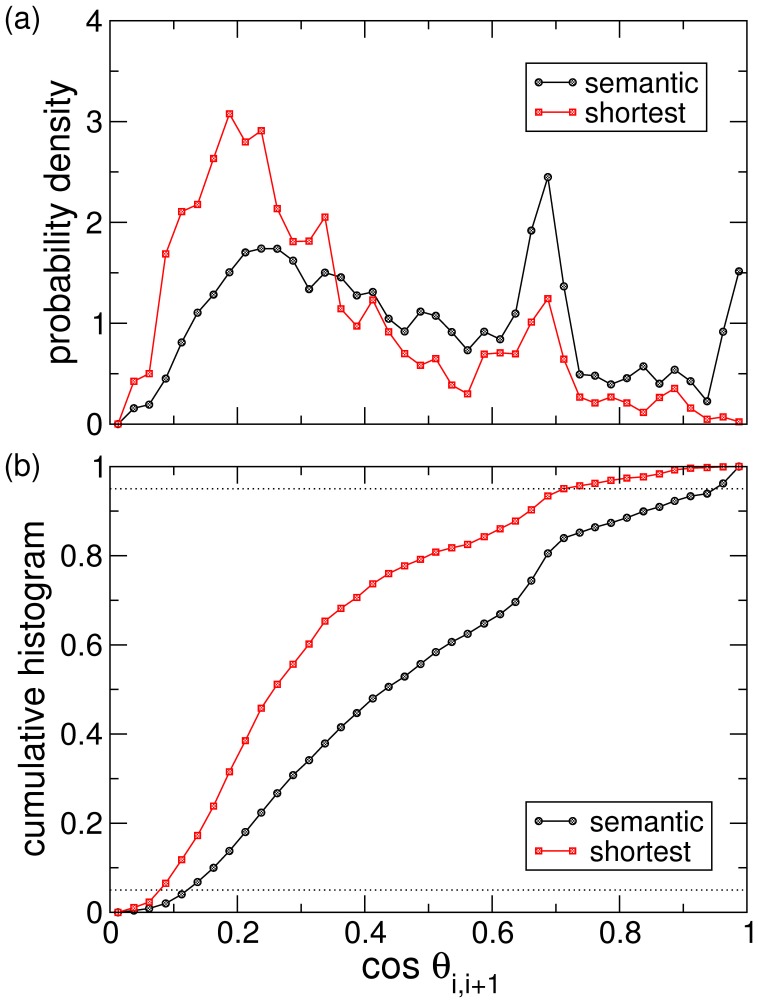
(a) Probability density of similarities between consecutive nodes along all semantic (black circles) and shortest paths (red squares). Semantic paths exhibit a peak around 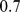, whereas the mode of the distribution for shortest paths is peaked around 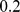. This fact shows that similarity between consecutive jumps from source to target along semantic paths is smooth, whereas similarity can change abruptly along shortest paths. (b) Cumulative probability of similarities between consecutive nodes. Note that the distribution for semantic paths lays below the shortest paths’ one. Dotted lines mark the 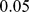 and 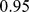 probability levels.

The question remains, however, as to how many steps of the random walk should we take, i.e., which should be the value of 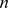. To solve this point, it is important to remind that the random exploration process is triggered to collect information about the underlying topology. The walker should have at least the chance to visit the whole network. This implies, in practice, that the process is able to connect the two furthest nodes in the network, i.e. 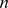 must be greater or equal to the diameter 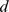 of the network. This diameter scales, in the case of scale-free complex networks as 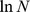 [7]. In our case-study network, the Simple English Wikipedia, results for RIM are obtained using 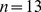 according to the observed diameter of the network.

RIM fits naturally in the family of path-based similarity measures [28–4]. The distinctive feature of RIM is that two nodes are similar if random walkers departing from them behave similarly. The information of the navigation process is stored in vectors, whose projections give a similarity measure between nodes.

**Table 2:** Word-pair semantic similarity measurement.

*Word pair*	*Human* [43]	α = 0	α = −0.5	α = 0.5
car-automobile	3.92	1.000	1.000	1.000
gem-jewel	3.84	1.000	1.000	1.000
coast-shore	3.7	0.548	0.313	0.702
magician-wizard	3.5	0.369	0.103	0.577
food-fruit	3.08	0.656	0.249	0.833
bird-crane	2.97	0.728	0.291	0.911
brother-monk	2.82	0.369	0.354	0.572
cemetery-woodland	0.95	0.117	0.033	0.360
food-rooster	0.89	0.622	0.083	0.902
coast-hill	0.87	0.377	0.051	0.651
forest-graveyard	0.84	0.188	0.061	0.451
shore-woodland	0.63	0.270	0.085	0.572
monk-slave	0.55	0.447	0.128	0.750
coast-forest	0.42	0.451	0.208	0.683
chord-smile	0.13	0.114	0.020	0.432
glass-magician	0.11	0.199	0.035	0.501
noon-string	0.08	0.232	0.050	0.486

We used the subset of pairs provided in reference [43] (Human judgment column), and reproduced for comparison purposes in reference [42], that are found in the giant component of SEW. RIM cosine similarities are listed for three different weighting schemes parameterized by 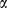 (see Eq. (5)).

**Table 3:** Pearson’s correlation coefficients between similarity ratings and the average ratings reported by Miller and Charles [43] for the subset of pairs listed in Table 2.

*Similarity method*	*Correlation*
Edge based	0.554
Node based	0.763
Combined distance	0.834
α = 0	**0.736**
α = −0.5	**0.727**
α = 0.5	**0.606**

For the sake of comparison, we include the correlation coefficients obtained by Jiang and Conrath [42] for the three similarity schemes (edge based, node based and combined distance) studied in that reference. Note that these schemes are based on word classifications provided, for example, by WordNet. The node-based scheme evaluates the similarity between two concepts as the maximum similarity score among all the classes that subsume simultaneously both concepts. The edge-based distance approach estimates the distance (edge length) between nodes which correspond to the concepts being compared. The combined approach is derived from the edge-based notion by adding information content (as in the node-based scheme) to edge weights.

### A Networked View of the Simple English Wikipedia

Our algorithm for navigation is a general-purpose method, as long as data can be modeled as a network, nodes representing meaningful entities (words, expressions, etc.) and links standing for content-related relationships (“is-a”, “is-part-of”, etc.). A perfect example of these generality can be found in Wikipedia, where links between articles stand for many types of relationships. For instance, the Wiki entry for Andréy Markov in the English Wikipedia has links to Russia (place of birth), Mathematics (the most general framework of his contributions), many people he interacted with, etc. For this reason we have chosen the complete Simple English Wikipedia (SEW) to test our proposal. In practice, we build the SEW network by linking a pair of nodes 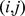 if 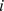 –an entry in SEW– contains an internal link to 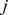.

The SEW database presented here corresponds to the dump of March 27, 2011. We only consider meaningful internal links, i.e., we filter out redirects and disregard any external links. Links to other Wikipedia resources –images, edition information, etc.– are disregarded as well [35]. After that pre-processing, the resulting network is formed by 68,558 articles (nodes), but not all of them are accessible, i.e., there exists a minority of articles which point to other nodes but are never pointed at. Given that our measures will be systematically compared to shortest paths, we ensure the existence of such paths by extracting the strongly connected giant component, which comprises 54,526 nodes and 2,313,665 directed links.

Pages in SEW have an average number of out-going connections 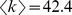, which means that the network is very sparse. In fact, the density of out-going connections is four orders of magnitude smaller than the linkage density expected for a fully connected network without self-loops and with the same number of nodes. This topology exhibits a rich local structure, with a clustering coefficient 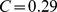, and despite its large size the average shortest path length is 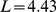. The most distant articles in SEW lie at a distance of only 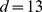 (diameter). In conclusion, SEW fits properly in the well-known concept of “small-world” network [36]. Furthermore, it exhibits a long-tailed in-degree distribution, which implies the existence of hubs –nodes which are richly connected [37].

Links in this networked view of SEW are unweighted. However, RIM demands that link strengths must be normalized. Given this situation, one may define the transition probability matrix 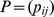 as 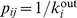 for all 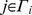 (i.e., for all of its neighbors), 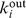 being the number of hyperlinks that a SEW document contains (its out-degree). However, this implies that a random walker will move from a node to any of its neighbors with equal probability, which is at odds with the evidence that not every piece of information is equally important. We use here the approach presented in Eq. (3) that can be easily extended to directed networks,
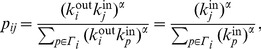
(5)where 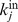 is the number of Wikipedia articles pointing at article 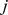 (its in-degree). Note that this framework generalizes the simplest scheme (uniform transition probabilities), which is recovered in the case 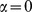. In the case of 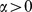, the walker will prefer visiting nodes of large degree. Negative values of 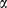 will bias the random walker towards nodes with lower connectivity.

The kind of biased random walks that we use in this contribution can be regarded as a local approximation of optimal random walks [38]. Maximal-entropy rate random walkers are defined by transition probabilities such that the walkers are maximally dispersing in the graph, exploring every possible path with equal probability. On correlated networks, maximal-entropy random walks can be obtained by considering a random walk whose motion is biased as a power of the target node degree, as in our case. Therefore the choice of biased random walkers ensures an efficient exploration of the network. A similar (and complementary) approach to the one followed here would consider biased walks as unbiased ones on weighted graphs, where dynamical flows are embedded into link weights [39].

## Results

We have implemented and tested our approach on the SEW data. The analysis we have developed tries to reveal the validity of the approach to complement any web search engine, recommendation system or information discovery technique. We restrict ourselves to make use only of local information on the similarity map. Although our method is completely general, we will focus on the semantic aspects of navigation over networks since our case-study dataset involves language. The advantages of having a semantically-coherent path of words become apparent in the design of efficient recommendation systems, web tagging methods and information retrieval algorithms.

### Navigation

The navigation method we propose is strictly guided by the underlying map of similarity relationships obtained from RIM. The defining aspects of the navigation algorithm are its being deterministic, using a greedy strategy and being self-avoiding. It is deterministic in the sense that the navigation process will either reach its target or it will fail. When the process gets stuck, that navigation trial aborts. Greediness means that the algorithm always seeks the best option to jump to, i.e. starting from the source node, the search process jumps to the node in its neighborhood with highest similarity to the target. Note that the algorithm yields a non-monotonic approach to the target, because it is possible that the next-hop node has a lower similarity to the target than the current one. Self-avoidance helps the process not to get trapped into endless cycles.

A suitable semantically-sensitive path must reach a compromise between the richness of the information it provides and the length cost it represents. Too long semantic paths become inefficient. Moreover, a local-based algorithm, i.e., one that relies only in information from its nearest neighbors, may fail to accomplish every possible path in a network.

Given these constraints, we present in the first place results concerning success and cost, regardless of content. The success rate is defined simply as the fraction of successful chains (paths that reach the target web page). The path cost is defined as 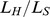, where 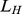 is the length of the path from the source to the target obtained with the heuristic local semantic navigation; and 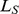 is the length of the path from the source to the target obtained using the shortest path (global information). On the SEW network, we selected 100 articles as targets and attempted to construct paths between any possible source and these targets. This means that over 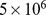 paths have been attempted. For the sake of completeness, the choice of target nodes has not been made at random. On the contrary, we have measured for each node in the network a centrality value (the *coreness* or 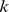 *-core* of each node [40]), which classifies nodes as belonging to different levels or shells, from the core to the periphery of the network. Examining this quantity enables us to choose heterogeneous target nodes which belong to distinctly connected parts of the topology. Since the 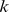-core is positively related to degree, choosing nodes with a wide range of 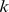-core ensures that they also exhibit heterogeneous total degree 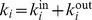. Targets have been chosen so as to guarantee the presence of both peripheral and core shells. Admittedly, other than this topological classification, targets have been chosen arbitrarily.

Figure 1 depicts, for different weighting schemes (i.e. as a function of 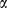), both the global average success rate (upper panel) and global average cost (lower panel). Remarkably, 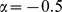 yields optimal results regarding both concepts, with over an 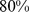 of success rate and average 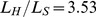. Given the simplicity of our navigation heuristics, our success rate should be compared to that of Milgram’s experiment [9] and the routing proposed by Boguña et al. [2], who reached success rates of around 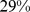 and 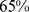, respectively. It is worth mentioning that optimal results are obtained for 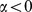. We interpret this as the fact that systematically favoring hubs (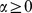) diminishes the capacity of random walkers to explore local neighborhoods of sparsely connected nodes, thus semantic relations can not reflect the rich modular structure of the network. A negative 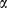, instead, forces the diffusive dynamics to remain trapped for some time in these semantically rich substructures.

Admittedly, the retrieval of content-sensitive chains seems to have a downside: the average cost of semantic paths triples that of shortest paths. Nonetheless, it is worth noticing results in Figure 2. In the figure we show, for different weighting schemes and within successful source-target navigations, the proportion of paths at cost 1, 2 and so on. Note the logarithmic scale in the 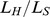 axis. Significantly, for the optimal case 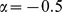 (in black circles), over a 75% of successful chains have 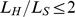, the global average being increased due to a minority of chains with large cost.

We now turn to which targets (out of the 100 preselected) exhibit better behavior when it comes to navigating towards them. As expected, Wikipedia articles with high accessibility (large 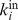) are reachable from almost anywhere in the network. Figure 3 illustrates this conclusion very clearly, both regarding success rate (upper panel) and cost (lower panel): nodes with 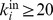 have perfect behavior (100% success, 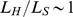), with few exceptions. This is true both for the optimal weighting scheme (black circles) and for the unweighted case (red squares).

Table 1 samples some chains to compare performance between shortest and similarity paths. For each pair of SEW pages, we first list the path following our proposed heuristics, then the shortest path. By visual inspection we observe that shortest paths frequently yield conceptual gaps between contiguous words, whereas our heuristic path provides a smooth trajectory in the semantic space, jumping between concepts whose semantic similarity is apparent.

Figures 4 and 5 try to picture the navigational paths displayed by both methods. The first figure (Thermodynamic_State 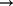 Seminar) is an example of optimal efficiency of our heuristic navigation, since 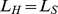. Additionally, successive steps in the semantic path have closer similarities to the target word than shortest-path steps. The second figure (Carlsberg 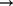 Sega_Game_Gear) illustrates how a suboptimal heuristic navigation attempt (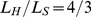) is compensated by a coherent path in terms of meaning. At some point, shortest paths move to a “semantically unrelated” node which acts as a hub, providing an efficient –though semantically poor– shortcut towards the target.

In order to provide a quantitative measure of the degree of smoothness that Table 1 and Figures 4 and 5 show, we have calculated the histogram of similarities between all pairs of consecutive words along paths and compared it with the same histogram for shortest paths. Results are shown in Figure 6. We have used 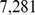 semantic paths between pairs of our preselected words from a subset of 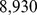 paths (notice that not every navigation attempt is able to reach the target) to obtain the corresponding histogram. On the other hand, there are up to 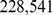 shortest paths for the same set of preselected pairs, because most of them are strongly degenerated (average degeneracy is 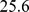). The probability distributions depicted in Figure 6a exhibit global maxima at similarities around 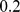 (shortest paths) and around 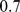 (semantic paths). This confirms quantitatively that similarities along semantic paths are smoother than for shortest paths, in accordance with the abrupt changes observed in the samples shown in Table 1 and Figures 4 and 5. The maxima of semantic paths does not occur, however, at similarities close to 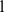. Note that the similarity between consecutive nodes should not necessarily be monotonically increasing, since navigation chooses the most similar neighbor *to the target* from the set of available ones, i.e., those not yet visited.

More formally, the cumulative distribution of the similarity jumps in heuristic paths is systematically smaller than that of shortest paths (see Figure 6b). This means that consecutive nodes in heuristic paths are “statistically more similar” than those of shortest paths –according to the well-known criterion of first-order statistical dominance [41].

### Performance of the Similarity Measure

We finally assess the semantic validity of the similarity map by comparing our similarity measure with a benchmark in Natural Language Processing. Jiang and Conrath [42] proposed a similarity measure which was successfully confronted to a set of words whose similarity, in its turn, was previously assessed by human judgment by Miller and Charles [43]. Human similarity ratings were tabulated for a set of 30 noun pairs, and later Jiang and Conrath used that set of pairs to validate their similarity measure. Note that this comparison is unfavorable to highlight our performance in several ways: i) Jiang and Conrath similarity measure is based on the taxonomy provided by WordNet [44], hence such a measure already incorporates human knowledge in its definition, whereas our source of information is purely topological and no taxonomies are predefined, ii) the structure of WordNet is not even similar to the connectivity in SEW, and iii) the number of words in WordNet is approximately 20,000 words larger than SEW. Even in this hard scenario, our approach shows to be competitive in semantic content. In Table 2 we present the subset of words in the intersection of SEW and the experiment by Miller and Charles [43], and the corresponding similarity at different values of the parameter 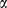 in the weight of links (c.f. Eq. (5)). The correlation values between the similarity ratings and the mean human ratings reported by Miller and Charles are listed in Table 3. Note that the correlation obtained is only a 10% lower than that obtained by Jiang and Conrath.

## Discussion

In summary, we have proposed a general and extensive method to construct a locally navigable map based on similarities of networked data. We have adopted a complementary vision of similarity between networked objects that emerges solely from its relative position in a network. We developed the idea that nodes that *see* the network the same way are themselves similar. The process used to explore the network from any node is based on random walkers that keep track of visits to other nodes. The view that every node has of the entire network (i.e., the set of feature vectors) is transformed into a map using the cosine projection. This map is the underlying structure used for local semantic navigation, based on searching for the neighbor that is more similar to the target. Note that although we need global information of the network to build up the similarity map, semantic navigation proceeds locally. Previous works aimed to network exploration have been inspired by similar ideas and are based solely on local information [45].

In terms of efficiency, our algorithm’s bottleneck is the calculation of the similarity matrix [see Eq. (2).] The computation of feature vectors [matrix (4)] is not so demanding provided that the original transition probability matrix 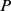 is sparse. The computational cost of 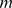 feature vectors is of order 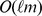, 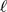 being the number of links and 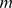 the number of nodes of the network. The computation of the similarity map involves 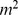 entries, each one of them being a scalar product, which in its turn increases time complexity by a factor of 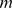. Consequently, the overall time complexity of our method is 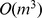.

For practical purposes, the similarities between nodes can be calculated as navigation proceeds. We simply need to store all the feature vectors and calculate, for node 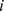, the cosine of each 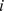
‘s neighbor with the target node. For large networks, both algorithms (i.e. the derivation of the map and the navigation procedure) are easily scalable and efficient using linear algebra parallel computations.

We have validated our approach confronting its outcome with human ratings of similarity between words extracted from the original, WordNet-based, reference of Jiang and Conrath [42]. Even in this disadvantageous scenario –WordNet is an annotated taxonomy with explicit semantic relationship coding– our purely topology-based algorithm provides correlations with human semantic judgment comparable to Jiang and Conrath’s similarity measures.

We have tested our algorithm’s performance in terms of path lengths compared to shortest-path lengths. The results are encouraging and the semantic smoothness of the paths, remarkable. The similarity map proposed in this paper can be readily employed to support many semantic and social web applications, such as tagging and recommendation. Another straightforward application of the local semantic navigation proposed here is to enrich web search and navigation for knowledge exploration. Finally, it is our guess that users would be more effective in performing an exploration or learning task by following semantically-coherent paths instead of shortest-length paths.
